# Potential Role for the Metnase Transposase Fusion Gene in Colon Cancer through the Regulation of Key Genes

**DOI:** 10.1371/journal.pone.0109741

**Published:** 2014-10-15

**Authors:** Panagiotis Apostolou, Maria Toloudi, Eleni Kourtidou, Georgia Mimikakou, Ioanna Vlachou, Marina Chatziioannou, Vasiliki Kipourou, Ioannis Papasotiriou

**Affiliations:** Research Genetic Cancer Centre Ltd (R.G.C.C. Ltd), Filotas, Florina, Greece; University of Navarra, Spain

## Abstract

The Metnase fusion gene consists of a SET histone methyltransferase domain and a transposase domain from Mariner transposase. This transposable element is involved in chromosome decatenation, enhances DNA repair, promotes foreign DNA integration, and assists topoisomerase II function. This study investigates the role of Metnase in colon cancer homeostasis and maintenance of the stemness phenotype in colon cancer stem cells (CSCs). Silencing of Metnase was performed in human cancer cell lines before and after treatment with cisplatin, and in colon CSCs. Subsequent changes in the expression of genes involved in repair mechanisms, DNA synthesis, topoisomerase II function, and metastasis as well stemness transcription factors were studied with RT-qPCR experiments. Cellular viability and apoptosis were evaluated by flow cytometry. The results suggest that Metnase influences the expression of many genes involved in the above processes. Furthermore, Metnase levels appear to impact upon expression of NANOG, OCT3/4, and SOX2. Suppression of Metnase also led to an increase in apoptosis. Therefore, Metnase may possess an important role in DNA repair, topoisomerase II function, and the maintenance of stemness during colon cancer development.

## Introduction

Metnase is a fusion gene with a SET histone methyltransferase domain and a Mariner transposase domain. Several of the main functions of HsMar1 transposase are shared with Metnase [1]. Metnase is a non-homologous end-joining (NHEJ) repair protein [2], and is involved in many cellular processes including mediation of foreign DNA integration, chromosome decatenation [3], and DNA repair [4] and replication [5]. Metnase further mediates resistance to topoisomerase II inhibitors through an interaction with topoisomerase (DNA) II alpha (TOP2A) [6]. These established roles in combination with recent experimental data suggest that Metnase may have a crucial role in cancer development and progression, which could be exploited during cancer treatment.

Colorectal cancer is the second leading cause of cancer in women, the third in men, and the fourth most common cause of cancer death overall [7]. The use of platinum-based chemotherapeutics is commonplace in treatment regimes. However, many patients either possess or develop resistance to these compounds [8]. Furthermore, cancer stem cells (CSCs) have the capacity for self-renewal and are resistance to chemotherapy and radiation treatment [9]. Therefore, improvements to current treatment strategies are required.

The present study examines the relationship between Metnase gene expression and colorectal cancer development. As transposable genetic elements are implicated in genome rearrangement, they may regulate many transcription factors. These factors could in turn regulate genes that are involved in resistance, metastasis, or apoptosis. An evaluation of the complement of genes that are affected by Metnase as well as their correlation with basic cellular activities may enhance our understanding of how Metnase influences cancer development. Such knowledge could also contribute to improvements in cancer treatment programs.

This study examines the expression levels of several genes important in cellular development and DNA synthesis and repair before and after knockdown of Metnase by siRNA. These genes were DNA excision repair protein (ERCC1), dipeptidylpeptidase IV (CD26), Met proto-oncogene (cMET), TOP2A, topoisomerase (DNA) II beta (TOP2B), thymidylate synthase (TYMS) and DNA (cytosine-5-)-methyltransferase 1 (DNMT1). The effect of Metnase silencing was also investigated in a colorectal cancer cell line following treatment with cisplatin. While oxaliplatin is mainly used in clinical settings, here we wished to investigate the role of Metnase in a resistant cell line. According to experiments that were previously performed in the HCT-116 cell line, we have found that more resistance mechanisms develop following treatment with cisplatin. Finally, a potential relationship between Metnase and maintenance of the stemness phenotype of colon CSC was investigated by silencing Metnase and measuring levels of NANOG, POU class 5 homeobox1 (OCT3/4), and SRY (sex determining region Y)-box2 (SOX2), all of which have crucial roles in the maintenance of stemness [10], [11].

## Material and Methods

### Cell Culture

Human colon CSCs (36112-39P; Celprogen, CA; USA) and HCT-116 Human colon carcinoma cells (91091005; ECACC, UK) were cultured in appropriate growth medium (M36112-39PS; Celprogen, and D5546; Sigma-Aldrich, Steinheim; Germany) supplemented with 10% FBS (10270-106; Gibco, NY; USA) in 25 cm^2^ flasks (E36102-29P-T25; Celprogen, and 430639; Corning, NY: USA) at 37°C in a 5% CO_2_ environment. HCT-116 cells were also treated with 1 µg/mL cisplatin (P4394; Sigma-Aldrich) for more than 10 passages and cultured in a similar manner.

### siRNA transfection

During the exponential growth phase, cells were plated in 24-well plates (E36112-39; Celprogen, and 831.836; Sarstedt, Nümbrect; Germany) and transfected with Metnase-specific siRNA using Lipofectamine 2000 Reagent (11668-027; Invitrogen, CA; USA), according to manufacturer's instructions. The siRNA (5′-UAAAACCUCACCAGCAUAUUU-3′) was designed in accordance with the rules of Reynolds et al. [12] and subjected to BLAST analysis to ensure specificity. Following 48 h incubation, cells were harvested by trypsinization (25200-072; Invitrogen). Vehicle-alone and non-specific siRNA controls were included. The mRNA knockdown was calculated relative to the non-targeting control siRNA in each experiment. Experiments were repeated three times in triplicate. The expression level of the gene of interest and percentage knockdown was calculated using the comparative Ct method:



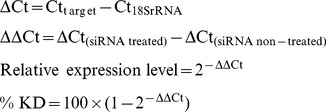



### Evaluation of cells

Cells were evaluated by cellular and molecular assays. Cellular assays were based on the ability of CSCs to form microspheres. The cultures have previously been evaluated by gene expression analysis of specific transcription factors, so authentication of cell lines was conducted by measuring the short tandem repeat profile and comparing with the manufacturer's profile. Cultivation of CSCs was conducted for over 30 passages to exclude the possibility of incorporating embryonic stem cells (ESCs) in the experiments, since CSCs are immortal unlike ESCs.

### Molecular Assays

RNA from cell cultures was extracted using the RNeasy mini kit (74105; Qiagen, Hilden; Germany). RNA samples were evaluated both spectrophotometrically and by agarose gel electrophoresis visualization of the 18S–28S bands. Genomic DNA was removed by using RNase-Free DNase (79254; Qiagen).Subsequently, 1 µg of this RNA was used as a template for cDNA synthesis with an iScript cDNA synthesis kit (1708891; Bio-Rad, CA: USA). Real-time PCR, was performed using the iTaq Universal SYBR Green Supermix (1725124; Bio-Rad) with each sample in triplicate. Specific primers for each marker and for an endogenous control gene (18S rRNA) were designed with Genamics Expression 1.1 software [13]–[16]. Primer sequences were analyzed by BLAST to exclude those who amplified undesired genes. Table 1 shows the sequences of the primers.

**Table 1:** Primer pairs that were used in qPCR.

Gene	Primer	Sequence (5'-3')	Accession No	Amplicon Length	Location	Splice Variant
18S rRNA	Forward	TGCCCTATCAACTTTCGATGGTAGTC	NR_003286	112bp	22p12	–
18S rRNA	Reverse	TTGGATGTGGTAGCCGTTTCTCA				
NANOG	Forward	TGAGATGCCTCACACGGAGACTG	NM_024865	138bp	12p13.31	Transcript variant 1
NANOG	Reverse	GGGTTGTTTGCCTTTGGGACTG				
OCT3/4	Forward	GGTGCCTGCCCTTCTAGGAATG	NM_001173531	97bp	6p21.31 exon 5	Transcript variant 3
OCT3/4	Reverse	TGCCCCCACCCTTTGTGTTC				
SOX2	Forward	CAACGGCAGCTACAGCATGATG	NM_003106	91bp	3q26.3-q27 exon 1	-
SOX2	Reverse	GCGAGCTGGTCATGGAGTTGTACT				
Metnase	Forward	GCAGCAGAAACAACTCGCAACATC	NM_001243723	123bp	3p26.1 exon 3	Transcript variant 2
Metnase	Reverse	ACGCTCCTCATCTTCAAGGCTCTC				
ERCC1	Forward	GCTACCACAACCTGCACCCAGACT	NM_001166049	152bp	19q13.32 exon 5	Transcript variant 3
ERCC1	Reverse	GCAGTCGGCCAGGATACACATCT				
CD26	Forward	GAGATGTTCCGGTCCTGGTCTG	NM_001935	127 bp	2q24.3 exon 16–17	–
CD26	Reverse	TTTGGAGGGCATCTGGACATTC				
cMET	Forward	AACAGGTGCAAAGCTGCCAGTG	NM_000245	95bp	7q31 exon 19	Transcript variant 2
cMET	Reverse	GCACGCCAAAGGACCACACAT				
TOP2A	Forward	TGGTCCTGAAGATGATGCTGCTATC	NM_001067	120bp	17q21-q22 exon 16	–
TOP2A	Reverse	GGAAGCCCAAGTAACTTTCGTTGTC				
TOP2B	Forward	CCCAAGAGAGCCCCAAAACAGA	NM_001068	151bp	3p24 exon 34	–
TOP2B	Reverse	CGCCTTCATTTTCAGAGCCAGAT				
TYMS	Forward	TCTGCTGACAACCAAACGTGTGTTC	NM_001071	123bpbp	18p11.32 exon 2	–
TYMS	Reverse	CCATTGGCATCCCAGATTTTCAC				
DNMT1	Forward	CTGGACGACCCTGACCTCAAATATG	NM_001130823	126bp	19p13.2 exon 16	Transcript variant 1
DNMT1	Reverse	CGCCTCATAACTCTCAAAGCCAGAC				

The PCR reaction program was as follows: initial denaturation at 95°C, 50 cycles of denaturation at 95°C for 10 sec followed by annealing at 59°C for 30 sec. A final extension step was performed at 72°C for 10 min followed by melting curve analysis. Data were analyzed according to the method of Livak and Schmittgen [17]. In all PCR reactions, appropriate controls were used. The positive control was cDNA from a Universal Human Reference RNA (740000-41; Agilent, CA; USA) and negative controls were no-template, no-enzyme controls as well as Human genomic DNA (G304A; Promega, WI; USA). Finally, a no-reverse transcription control was used in cDNA synthesis. The standard curves of all primers are presented in figure S1.

### Flow cytometry

Cells were stained with PE Annexin V and 7-Amino-Actinomycin (559763; BD Biosciences, CA; USA) for 15 min followed by resuspension in 0.5 mL sheath fluid (8546859; Beckman Coulter, Nyon; Switzerland) and flow cytometry analysis of more than 50,000 events. The data were analyzed with FCS Express Software (DeNovo). In each case appropriate positive and negative controls were used.

### Statistical Analysis

The quantitative polymerase chain reaction (qPCR) results were assessed according to the Kolmogorov-Smirnov test; all samples had normal distribution. Median values were used for the analysis. Mann-Whitney tests were also performed on the qPCR data [18], [19]. All experiments were performed in triplicate three times. A p value <0.05 was considered significant.

## Results

### Gene Expression

**Figure 1:**
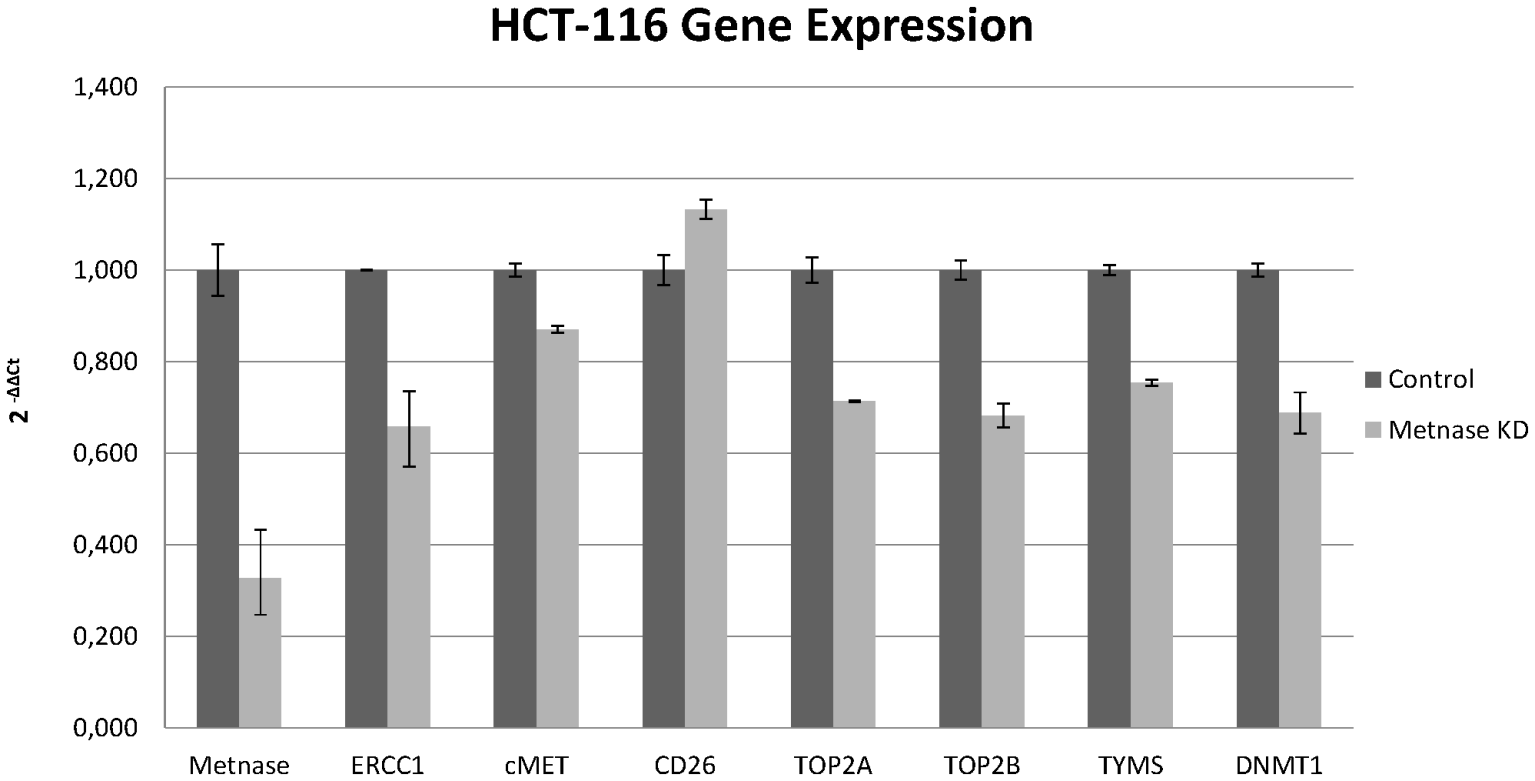
Metnase regulates gene expression in colon CSCs. Relative gene expression of transcription factors in Colon CSCs following Metnase knockdown. The percentage of Metnase knockdown reached 40%. The ΔΔCt method was used to perform the analysis. Each bar represents the average of the Ct values. The assays were performed in triplicate and a p-value <0.05 was considered significant. In the control sample the average value is 1.00 indicating that there is no change in gene expression. Values>1 indicate an increase in gene expression while values <1 indicate a decrease in gene expression. The conditions for subsequent experiments were the same.

**Figure 2:**
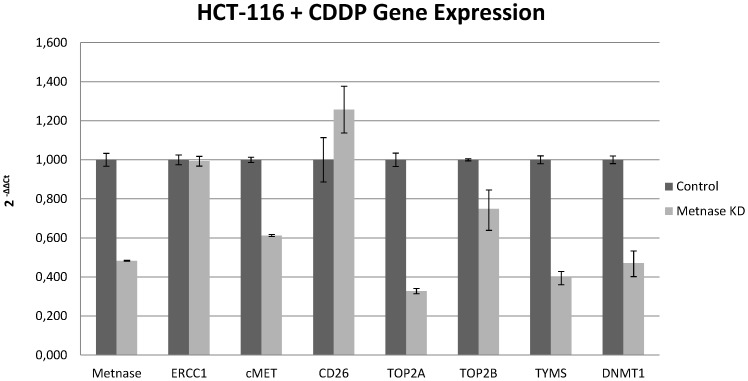
Metnase regulates gene expression in HCT-116 cell line. Relative gene expression of transcription factors in HCT-116 cells following Metnase knockdown. The percentage of knockdown reached 65%.

**Figure 3:**
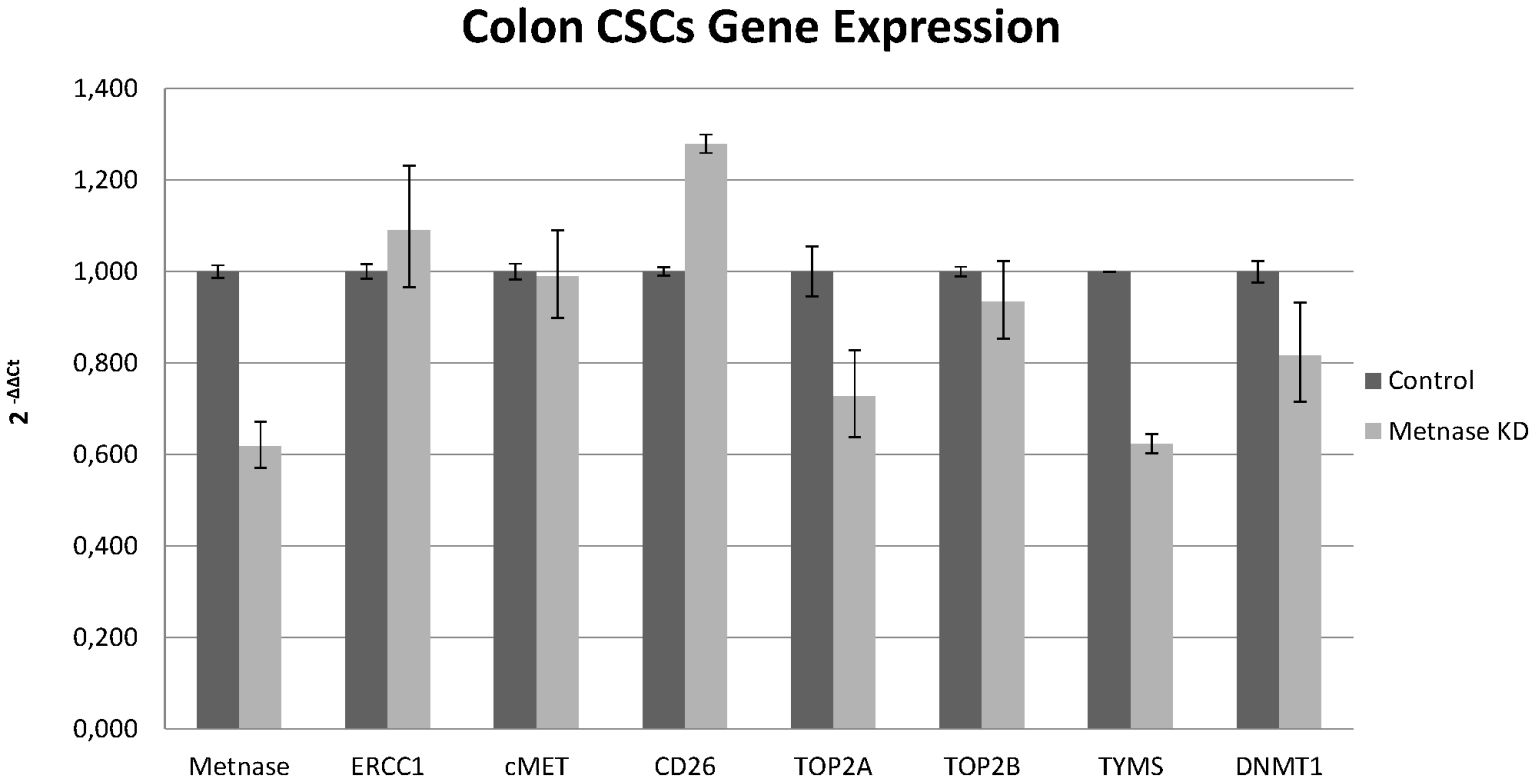
Metnase regulates gene expression in HCT-116 cells treated with cisplatin. Relative gene expression of transcription factors in HCT-116 cells treated with cisplatin, after Metnase knockdown. The percentage of knockdown reached 52%.

Silencing of Metnase expression by siRNA was up to 65% efficient in HCT-116 cells, 52% in HCT-116 cells treated with cisplatin, and 40% in colon CSCs (Figures 1–3). Suppression of Metnase in HCT-116 cells led to an increase in CD26 gene expression and a decrease in the expression of all other genes measured This decrease was higher for TOP2A, TOP2B, ERCC1, TYMS, and DNMT1, ranging from 25–35%, while a minor fall of 5–10% of cMET expression was observed (Figure 1). ERCC1 is involved in DNA repair processes, and its expression has been linked with sensitivity of this cell line to platinum compounds [20].

We observed similar results in HCT-116 cells treated with cisplatin. The enhanced decrease in topoisomerase II gene expression seen following Metnase silencing in HCT-116 cells treated with cisplatin compared with those not treated is also remarkable. TYMS and DNMT1 expression were decreased by up to 60%, while TOP2B expression was decreased by 18–35% and cMET by almost 40%. ERCC1 levels were not affected, indicating that treatment with cisplatin for many passages leads to development of resistance mechanisms (Figure 2).

**Figure 4:**
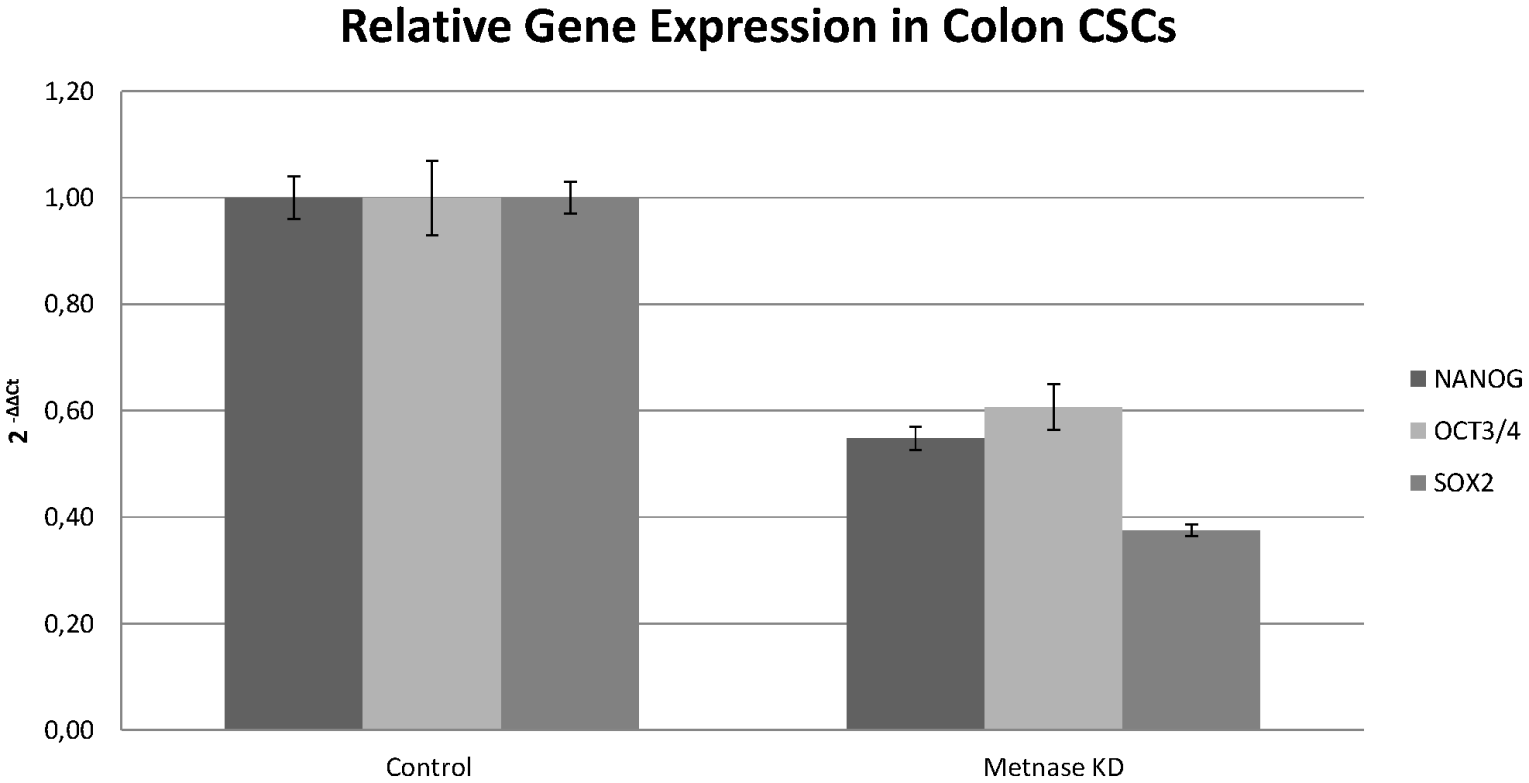
Metnase regulates gene expression of stemness markers. Relative gene expression analysis of the stemness transcription factors NANOG, OCT3/4, and SOX2 following Metnase knockdown.

Following silencing of Metnase in colon CSCs, the only gene measured that demonstrated increased expression was CD26. A decrease was observed for the TYMS and TOP2A genes, while the expression of ERCC1, cMET, and TOP2B appeared unaffected (Figure 3). These results demonstrated that CSCs are more resistant to chemotherapeutics than differentiated cancer cells. Overall, the expression levels of genes regarded as stemness transcription factors were decreased following silencing of Metnase expression in CSCs. This decrease in expression was up to 60% for SOX2, 40% for OCT3/4 and 45% for NANOG (Figure 4).

### Cell Viability-Apoptosis

The number of cells undergoing apoptosis as determined by flow cytometry was doubled following suppression of Metnase expression in both HCT-116 and HCT-116 + cisplatin cells. An increase in the number of dead cells was also observed. However, cellular viability was not significantly changed. In colon CSCs, cell viability decreased following Metnase silencing, but no change in the number of cells undergoing apoptosis was observed. The population of dead cells was higher in the HCT-116 cell line than in the other two. This could be attributable to the resistance mechanisms that develop in the cisplatin-treated cell line, or such mechanisms may natively exist in CSCs. Table 2 depicts data for each cell line.

**Table 2:** Percentage of dead cells and cells undergoing apoptosis before and after Metnase Knockdown.

	Cell line	Cells undergoing apoptosis (%)	Dead cells (%)
**HCT-116**	Control	2,03 (±0.20)	8,32 (±0.43)
**HCT-116**	Metnase Knockdown	4,01 (±0.35)	10,74 (±0.55)
**HCT-116 + CDDP**	Control	1,23 (±0.17)	2,77 (±0.22)
**HCT-116 + CDDP**	Metnase Knockdown	2,67 (±0.15)	3,96 (±0.30)
**Colon CSCs**	Control	1,9 (±0.15)	1,36 (±0.12)
**Colon CSCs**	Metnase Knockdown	1,55 (±0.26)	5,63 (±0.18)

## Discussion

The Metnase fusion gene methylates histone H3 at lysine 36 and possesses many characteristics of a transposase, including terminal inverted repeat sequence-specific DNA binding and DNA looping [21]. However, it cannot complete transposition reactions. Through its methylation activity, Metnase is implicated in DNA repair by the NHEJ pathway. This repair activity requires an interaction with Pso4 [22]. The ERCC1 protein is also involved through nucleotide excision repair [23]. This study suggests the existence of a relationship between ERCC1 expression and Metnase. Specifically, suppression of Metnase led to decreased expression of the repair gene, however, this decrease was less pronounced when cells were treated with cisplatin. This further supports a role for ERCC1 in cisplatin therapy.

TOP2A is the primary decatenation enzyme, resolving tangled or catenated chromatids [24]. This study also confirms that Metnase mediates resistance to topoisomerase II α inhibitors, with gene expression of TOP2A affected more than other genes following Metnase knockdown. This decrease was enhanced in cells treated with cisplatin and in CSCs. Furthermore, suppression of both TOP2A and TOP2B was observed, indicating that Metnase could be a target for combination chemotherapy with topoisomerase II inhibitors.

Expression of dipeptidyl peptidase IV (CD26) is correlated with colon cancer progression and CD26+ CSCs have been identified in human colorectal cancer. Here, CD26 gene expression was increased in all cases of Metnase knockdown. This enzyme is associated with immune regulation, signal transduction and apoptosis. This indicates that Metnase is involved in regulating apoptosis, perhaps through an interaction with CD26 [25].

The cMET proto-oncogene encodes the hepatocyte growth factor receptor and is closely associated with cancer development. Aberrant activation of cMET leads to tumor growth, angiogenesis and finally metastasis. In contrast to normal stem cells, CSCs express cMET, facilitating cancer persistence and spread [26]. Negative feedback regulation of MET-dependent invasive growth by Notch has been demonstrated in Drosophila [27], and Notch genes also regulate MET in humans [28]. However, this study found no association of cMET levels with those of Metnase transposase.

We observed a relationship between Metnase and two genes important in DNA homeostasis, TYMS and DNMT1. Gene expression of both was decreased in all cell lines following silencing of Metnase expression. TYMS generates thymidine monophosphate, which is subsequently phosphorylated to thymidine triphosphate for use in DNA synthesis and repair [29]. DNMT1 is an enzyme involved in the regulation of methylated cytosine residues, and its aberrant methylation is associated with cancer development [30]. This decrease in levels of TYMS and DNMT1 was enhanced in cells treated with cisplatin. Therefore, Metnase may be implicated in cancer development and establishment through interaction with or influence of several enzymes that possess key roles in cancer.

We also investigated the relationship between Metnase and transcription factors essential for maintaining stemness. CSCs are defined by the ability to self-renew, differentiate, and proliferate. CSCs express many transcription factor markers, but the most important are NANOG, OCT3/4 and SOX2 gene. NANOG is expressed in ESCs and has a key role in maintaining pluripotency. Its overexpression causes self-renewal in ESCs, while its absence leads to differentiation [31], [32]. To maintain stemness, the presence of two further transcription factors, OCT3/4 and SOX2 is required. OCT3/4 expression is also associated with an undifferentiated stage and self-renewal, forming a heterodimer with SOX2 and together these two proteins bind to DNA. SOX2 is a transcription factor essential for maintaining pluripotency, but its ectopic expression may be involved with abnormal differentiation of colorectal cancer cells [33], [34]. Knockdown of Metnase led to decreased gene expression of all transcription factors, indicating that Metnase may be involved in cancer establishment as well as in cancer development and progress.

Cellular viability appeared unaffected by Metnase knockdown. The cisplatin-treated cell line appeared to have fewer dead cells in compare with the non-treated cell line. Similar findings were obtained using colon CSCs. This further supports the resistance in chemotherapy observed in CSCs. However, Metnase silencing did impact upon the number of cells undergoing apoptosis. These could be explained by the development of alternative apoptosis-evading mechanisms developing in the CSCs compared with the treated cell line.

This study identifies that the Metnase fusion gene is heavily involved in DNA repair mechanisms, DNA synthesis, topoisomerase II resistance, apoptosis, and the maintenance of the stemness phenotype in colon cancer. Further studies are needed to elucidate the details of these interactions.

## Supporting Information

Figure S1 **Standard Curves – Standard curves for all primers used.** A: 18S rRNA, B: Metnase, C: ERCC1, D: cMET, E: CD26, F: TOP2A, G: TOP2B, H: TYMS, I: DNMT1, J: NANOG, K: OCT3/4, and L: SOX2.
(TIF)
