# Supplementary figures and images for: Potential Role for the Metnase Transposase Fusion Gene in Colon Cancer through the Regulation of Key Genes
Source: PLoS One. 2014 Oct 15;9(10):e109741. doi: 10.1371/journal.pone.0109741 (PMC4198141; doi:10.1371/journal.pone.0109741)

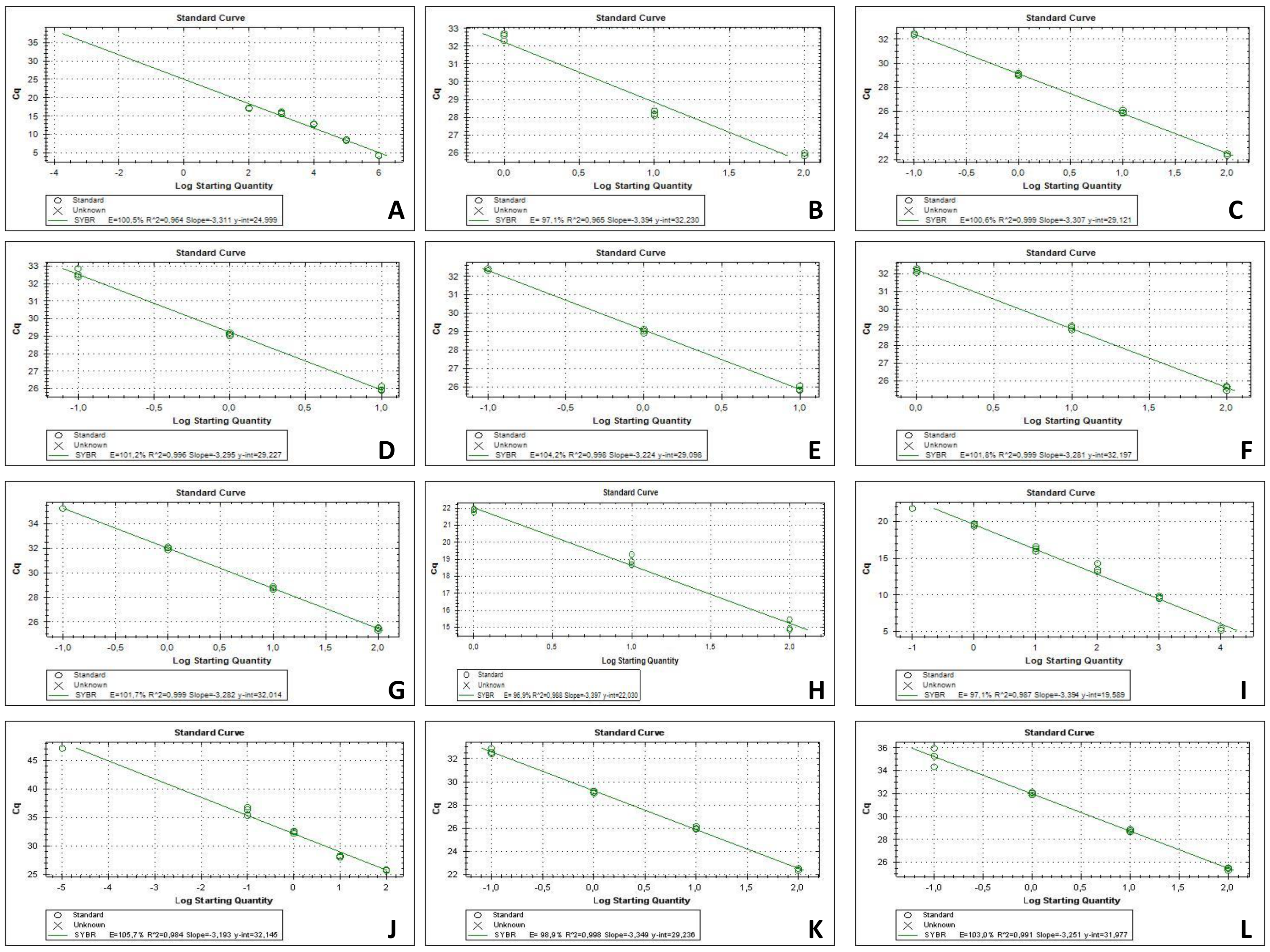

Supplement: Figure S1 — Standard Curves – Standard curves for all primers used. A: 18S rRNA, B: Metnase, C: ERCC1, D: cMET, E: CD26, F: TOP2A, G: TOP2B, H: TYMS, I: DNMT1, J: NANOG, K: OCT3/4, and L: SOX2. (TIF) [file pone.0109741.s001.tif]
